# Role of Bacterial Surface Components in the Pathogenicity of *Proteus mirabilis* in a Murine Model of Catheter-Associated Urinary Tract Infection

**DOI:** 10.3390/pathogens12040509

**Published:** 2023-03-24

**Authors:** Roman Herout, Sara Khoddami, Igor Moskalev, Alina Reicherz, Ben H. Chew, Chelsie E. Armbruster, Dirk Lange

**Affiliations:** 1 The Stone Centre at Vancouver General Hospital, Department of Urologic Sciences, University of British Columbia, Vancouver, BC V6H 3Z6, Canada; 2 Department of Urology, University Hospital Carl Gustav Carus, Technische Universität Dresden, 01062 Dresden, Germany; 3 Vancouver Prostate Centre, Department of Urologic Sciences, University of British Columbia, Vancouver, BC V6H 3Z6, Canada; 4 Department of Urology, Marien Hospital Herne, Ruhr-University of Bochum, 44649 Herne, Germany; 5 Department of Microbiology and Immunology, Jacobs School of Medicine and Biomedical Sciences, State University of New York at Buffalo, Buffalo, NY 14263, USA

**Keywords:** *Proteus mirabilis*, urinary tract infection, adhesion, invasion

## Abstract

*Proteus mirabilis* (*PM*) is a Gram-negative, rod-shaped bacterium that causes catheter-associated urinary tract infections (CAUTIs). The specific roles of bacterial surface components (BSCs) in *PM* pathogenicity and CAUTIs remain unknown. To address this knowledge gap, we utilized relevant in vitro adhesion/invasion models and a well-established murine model of CAUTI to assess the ability of wildtype (WT) and seven mutant strains (MSs) of *PM* with deficiencies in various genes encoding BSCs to undergo the infectious process (including adhesion to catheters) in both model systems. Overall, MSs adhesion to catheters and the different cell types tested was significantly reduced compared to WT, while no invasion of cells was evident at 24 h. In vivo, WT showed a greater number of planktonic (urine) bacteria, bacteria adherent to catheters, and bacteria adherent to/invading bladder tissue when compared to the MSs. Bacterial counts in urine for PMI3191 and *waaE* mutants were lower than that for WT and other MSs. The complementation of mutated BSC genes resulting in the biggest defects restored the invasion phenotype both in vitro and in vivo. BSCs play a critical role at various steps in the pathogenicity of *PM* including adhesion to indwelling medical devices and adhesion/invasion of urinary tissue in vivo.

## 1. Introduction

*Proteus mirabilis (PM)* is a Gram-negative bacterium first described by Gustav Hauser in 1885. He named this genus of Proteobacteria after the Greek god Proteus due to the bacteria’s changeability in form as it alternates between an immobile and a mobile, so-called “swarming” condition [1]. *PM* possesses the enzyme urease that splits urea in urine into carbon dioxide (CO_2_) and ammonia (NH_3_), which results in a rise in urinary pH [2]. Consequently, salts precipitate as a result of reduced solubility, resulting in the crystallization and subsequent crystal formation of (MgNH_3_PO_4_) and apatite (CaPO_4_) and eventual catheter encrustation and struvite stone formation [3]. Biofilm formation and catheter encrustation, both hallmarks of *PM*-induced catheter-associated urinary tract infections (CAUTIs), are financially and physically burdensome outcomes of urinary catheter use [4]. The first step in *PM* pathogenicity in the urinary tract is colonization, as the microorganism can attach to epithelial cells with various fimbriae and adhesins and potentially internalize within these cells [5]. Various virulence factors of *PM* have been described: fimbriae are outer-membrane pili that extend from the bacterial surface and usually facilitate adherence to surfaces, of which the chaperone-usher fimbriae are linear, unbranching, outer-membrane pili produced by Gram-negative bacteria that mediate their form of secretion and assembly [6,7]. *PM* HI4320 encodes 17 chaperone-usher fimbrial operons, and these fimbriae are regarded as key factors in adhesion and bacterial aggregation for *PM* in vivo [8,9]. To date, not much is known regarding the role of specific cell surface components in the adhesion to and invasion of uroepithelial cells and the overall pathogenicity of *PM* [10]. To begin addressing this gap in knowledge, we made use of transposon and Targetron mutants of genes involved in the synthesis of *PM* cell surface components, generated through previous studies, to identify *PM* HI4320 fitness factors, to assess their role in PM pathogenicity with specific emphasis on key adhesion and invasion processes [11]. These include transposon mutagenesis of *ucaJ*, *fim14C*, *PMI3191*, *arnA*, *pbpC*, and *waaE*.

Urothelial cell adhesin (UCA) frimbriae are adhesion proteins that have been observed to play a critical role in the attachment to the urothelium and are related to the K99 fimbriae of enterotoxigenic *E. coli* [12]. MrpJ is a transcriptional regulator encoded at the 3′ end of the mrp fimbrial operon in *PM*. Overexpression of MrpJ decreases flagellum-mediated motility in *PM* in order to regulate the opposing processes of motility and adhesion. UcaJ is one of 14 paralogues of MrpJ found in *PM* and functions as an inhibitor of motility when attachment is indicated [13]. *Fim14C* (PMI2998) is the major pilin subunit of a putative chaperone usher fimbrial operon, and *fim8F* (PMI1464) is part of another putative chaperone usher fimbrial operon [14,15]. *PMI3191* encodes for a glycosyltransferase that has been shown to contribute to *PM* lipopolysaccharide (LPS) [16]. *ArnA* (PMI1045) is the name of a gene that encodes for the bifunctional polymyxin resistance protein ArnA that physiologically catalyzes the oxidative decarboxylation of UDP-glucuronic acid to UDP-4-keto-arabinose, which is important in LPS formation [8]. This protein is also a significant contributor to bacterial resistance to antimicrobial peptides, as well as resistance to antibiotics, including polymyxin [17]. The penicillin-insensitive transglycosylase gene *pbpC* (PMI1850) encodes for a protein that is involved in the pathway peptidoglycan biosynthesis with regard to cell wall formation. The penicillin-binding protein 1C (pbpC) is one of several in *PM* HI4320 and represents a conserved feature across Gram-negative bacteria [18,19]. The gene *waaE* encodes for an LPS core biosynthesis glycosyl transferase, an enzyme required for LPS biosynthesis. This outer membrane component of Gram-negative bacteria is critical for antimicrobial peptide resistance, as well as swarming motility [20,21].

Collectively, the different genes being targeted are involved in the production of cell surface components with different functionalities, providing a broader overview of bacterial surface structures critical for *PM* adhesion and invasion of bladder epithelial cells and the overall infectious process. The identification of these key components represents a critical first step towards the development of targeted therapies to block key steps in the pathogenicity of *PM* and prevent associated morbidity and mortality.

## 2. Materials and Methods

### 2.1. Bacterial Strains and Growth Conditions

The studies were carried out using wildtype *PM* HI4320, a clinical isolate from the urine of a female nursing home patient with a long-term indwelling catheter (≥30 days), [22,23] along with 6 isogenic mutants of this same strain (PMI0532, PMI1045, PMI2998, PMI3191, PMI1850, PMI1464) (Table 1) generated as part of a previous study [24]. Briefly, the affected gene was disrupted by the insertion of a transposon carrying the kanamycin resistance cassette. A seventh isogenic mutant in *waaE* (PMI3136) was generated for this study via the TargeTron method: a kanamycin resistance cassette was inserted into the gene of interest, following the Sigma TargeTron group II intron protocol, as previously described [24]. In addition, two complemented strains (*cPMI3191* and *cwaaE*) were generated. Briefly, complementation vectors were constructed by amplifying the indicated gene along with approximately 500 bp of flanking sequence and ligating the amplified sequences into a linearized pGEN-MCS vector. Successful complementation was verified by selection on ampicillin plates and by PCR.

For bacterial culture, bacterial strains were separately cultured from freezer stocks (−80 °C) in fresh Luria broth (LB) media (10 g/L tryptone, 5 g/L yeast extract, 0.5 g/L NaCl) ± kanamycin or ampicillin. Strains were incubated in a shaking incubator at 225 RPM and 37 °C overnight. The following day, all cultures were sub-cultured in fresh media (LB, DMEM, or RPMI depending on the experiment) and left for a subsequent overnight subculture under the same conditions prior to use for experiments.

### 2.2. Catheter Adhesion Studies

To assess the potential for *PM* WT and mutants to interact with the catheter surface and form biofilm over time, an adhesion assay using 24G polyurethane (PU) catheters to be used for eventual in vivo analyses was performed in artificial urine. *PM* wildtype and the 9 mutant and complemented strains were separately cultured from freezer stocks in fresh LB media. Strains were incubated at 37 °C overnight. The following day, all cultures were sub-cultured in fresh LB media and left overnight in the same conditions. Bacterial stock solutions of approximately 1 × 10^8^ CFU/mL were prepared in artificial urine. The artificial urine was prepared as described by Brooks and Keevil [25].

Sterile catheter pieces were placed inside sterile Eppendorf tubes containing artificial urine and one of the 10 *PM* strains, and tubes were placed on a rotator and placed inside an incubator for either 4 or 24 h at 37 °C. At each timepoint, catheters were removed and washed gently in sterile PBS to remove any loosely adhered bacteria. Catheters from each strain were transferred to tubes of fresh PBS, sonicated, vortexed, serially diluted, and plated on LB agar for CFU counts. Agar plates were incubated overnight at 37 °C until bacterial colonies were visible, and distinguishable CFU counts between 2 to 50 colonies were documented.

### 2.3. Adhesion and Invasion Assays

For adhesion and invasion assays, three different human cell lines were used, including the kidney epithelial cell lines A498 and HEK293 and the human bladder epithelial cells line T24. Cells were cultured from liquid nitrogen freezer stocks. A498 and HEK293 cells were grown in Dulbecco’s Modified Eagle Medium (DMEM, Gibco™, ThermoFisher, Whaltam, MA, USA) and T24 cells with Roswell Park Memorial Institute (RPMI; Gibco™, ThermoFisher, Whaltam, MA, USA) 1640 media, all supplemented with 10% fetal bovine serum (FBS). Once cells had stabilized in their growing patterns and were 80% confluent, they were detached using Trypsin/EDTA(Gibco™, ThermoFisher, Whaltam, MA, USA). Cells were counted using an automatic cell counter and 1 × 10^4^–1 × 10^5^ cells were seeded in each well of a 24-well tissue culture plate. Cells were incubated at 37 °C and 5% CO_2_ and allowed to grow until 80% confluent. On the day of the experiments, two of the wells were trypsinized to quantify the number of cells per well and the average cell number used to determine the number of bacteria to be added to each well. Cell media were removed, and all cells were gently washed thrice with sterile PBS. Next, each of the bacterial strains to be tested was added at a multiplicity of infection (MOI) of 1:100. The plates were gently centrifuged at 500× *g* for 5 min to bring bacteria in close proximity to the epithelial cells without forcing them to directly interact. Plates were then incubated at 37 °C and 5% CO_2_ for either 2 h or 24 h.

To assess bacterial adhesion, bacterial cell media were removed after 2 h of incubation, and the monolayers were washed thrice gently with sterile PBS to remove any loosely adherent bacteria. Cells were then lysed using 0.1% Triton-X solution for 15 min at room temperature. The resultant cell lysates were then serially diluted in sterile PBS and plated on LB agar plates followed by overnight incubation at 37 °C and CFU counting the following day. Counts between 2 and 50 visible colonies were recorded and used for bacterial quantification.

To assess bacterial invasion, the media were removed and cells were washed thrice with sterile PBS at the 2 h and 24 h timepoints. Cells were then incubated in fresh media containing 1% Penicillin-Streptomycin for an additional 4 h at 37 °C and 5% CO_2_ to kill adherent bacteria. Next, cells were washed with PBS and lysed with 0.1% Triton-X at room temperature for 15 min. Internalized bacteria were quantified via CFU counts as described above.

### 2.4. LPS Gel Electrophoresis

To verify the complementation of genes affecting LPS synthesis, we extracted bacterial LPS via hot aqueous-phenol extraction followed by separation by SDS-PAGE (15%) (Supplementary Figure S1). *PM* WT and mutants were grown in 5 mL LB and incubated at 37 °C overnight. The culture was diluted 1:10 with LB, and a 1.5 ml suspension was created to an OD_600_ of 0.5. The bacteria were centrifuged at 10,600× *g* for 10 min, the supernatant was removed, and the LPS extraction was started immediately. Bacterial LPS was extracted via hot aqueous-phenol extraction according to Davies et al. [26]. LPS was separated by SDS-PAGE run on a 15% SDS-polyacrylamide gel.

### 2.5. In Vivo CAUTI Model

#### 2.5.1. Ethics Statement

The study was conducted according to the guidelines of the Declaration of Helsinki and approved by the Institutional Animal Care Committee of The University of British Columbia (Protocol A17-0297).

#### 2.5.2. Catheter Modification

Under aseptic conditions, 24G angiocatheters (Terumo Surflash^®^ Polyurethane I.V. Catheter 24 Gauge x 3/4”, Cat. No. SRFF2419, Terumo, Vaughn, Canada) were temporarily removed from the needles, and a 4 mm piece was cut off using a sterile blade. The angiocatheter and small piece were then re-assembled back onto the needle tip. Prior to being used for the in vivo experiments, all catheters were gas-sterilized with ethylene oxide to further ensure sterility and avoid any contamination.

#### 2.5.3. Mouse Experiments

Mice used in these experiments were male C57Bl/6 mice (Harlan^®^) at 12 weeks of age. Mice underwent inhalational anesthesia with 3% isoflurane. Under ultrasound guidance, (Vevo 770^®^ High-Resolution Imaging System, VisualSonics, Bothell, WA, USA) a 4 mm piece of a modified 24G catheter was implanted into the mouse bladder [27]. The day following catheter implantation, all mice underwent anesthesia as described above, and a bacterial solution containing 5 × 10^5^ CFU/mL in 50 μL PBS of one of the strains to be tested was percutaneously injected into the mouse bladder lumen using a 30G needle. Following bacterial injection, all mice were maintained under anesthesia at 1% isoflurane for an additional hour to promote bacterial attachment to the catheter and epithelium and to avoid premature micturition. On day 3, animals were anesthetized as above and urine was collected using a 30G needle under ultrasound guidance for subsequent quantification of planktonic bacteria via CFU counts, as described above. Subsequently, all mice were euthanized on day 4. All catheters were removed from the mouse bladders and aseptically transferred to tubes containing sterile PBS. After gentle washing with PBS, catheter samples were sonicated at 50/60 Hz for 10 min in an ultrasonic water bath (No. 21811-820, VWR^®^) followed by vortexing for 30 s at high speed to promote detachment of bacteria from the catheter. The PBS-bacterial solutions were then serially diluted and plated on LB agar plates for CFU counts, as outlined above.

### 2.6. Statistical Analysis

Significance was assessed using one-way analysis of variance (ANOVA), a nonparametric Kruskal–Wallis test, and the Wilcoxon signed-rank test. *p*-values were two-tailed at a 95% confidence interval. The analysis was performed with GraphPad Prism (version 8) software (GraphPad Software, San Diego, CA, USA).

## 3. Results

### 3.1. Adhesion to Polyurethane (PU) Catheter Pieces

**Figure 1:**
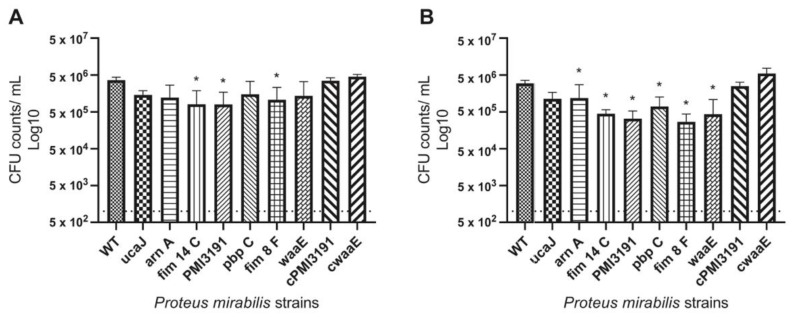
Adhesion of various *Proteus mirabilis* strains to 24G PU catheters in artificial urine. (**A**) Adhesion after 4 h. (**B**) Adhesion after 24 h. The data are represented as the mean ± SD of triplicate samples of three independent experiments. Statistically significant differences are indicated by an asterisk (* *p* < 0.05).

Adhesion at 4 h and 24 h was assessed to represent initial adhesion and colonization, respectively. Overall, all *PM* mutants showed similarly reduced adhesion to the catheter surface at the 4 h timepoint (Figure 1A), with the *fim14C, PMI3191* and *fim8F* mutants showing significant reduction. In terms of colonization, all mutants showed significantly reduced colonization levels at the 24 h timepoint (Figure 1B). Complementation of *PMI3191* and *waaE* returned adhesion and colonization levels to at least WT levels.

### 3.2. Adhesion and Invasion In Vitro

**Figure 2:**
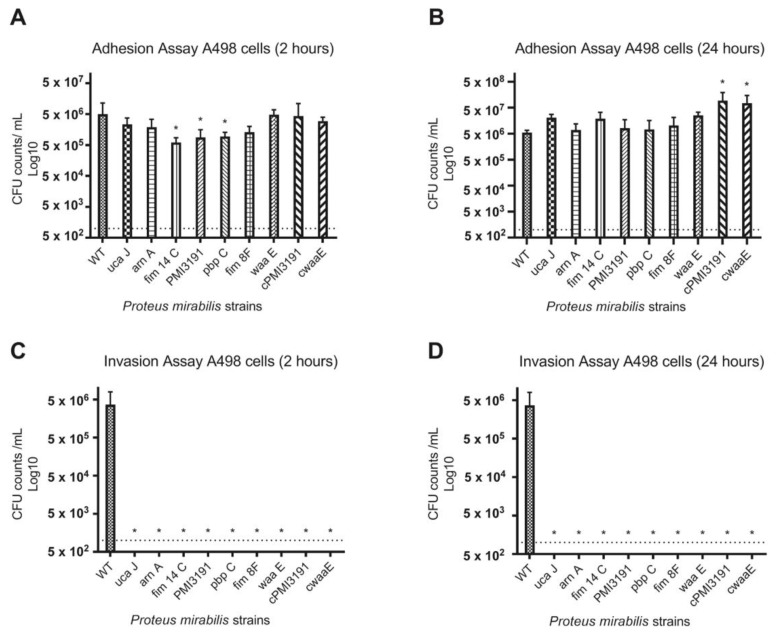
Adhesion and invasion of various *Proteus mirabilis* strains to and of, respectively, A498 kidney epithelial cells. (**A**) Adhesion after 2 h. (**B**) Adhesion after 24 h. (**C**) Invasion after 2 h. (**D**) Invasion after 24 h. The data are represented as the mean ± SD of triplicate samples of three independent experiments. Statistically significant differences are indicated by an asterisk (* *p* < 0.05).

We first tested adhesion and invasion in A498 kidney epithelial cells. At 2 h p.i., *PM* mutant strains *fim14C*, *PMI3191* and *pbpC* showed statistically significantly less adhesion to A498 cells when compared to WT (Figure 2A). However, these adherence patterns differed and showed some variance when observed at 24 h p.i. (Figure 2B). Many of the mutant strains had CFU counts comparable to or greater than that of WT (5.5 × 10^6^ CFU/mL) in the adhesion assay at 24 h. CFU counts for the complemented strains (*cPMI3191* and *cwaaE*) were significantly higher than WT counts. When quantifying invasion assay results at both 2 h and 24 h, WT invasion was 3.7 × 10^6^ CFU/mL, and there was no quantifiable invasion of the *PM* mutant strains at either timepoint (Figure 2C,D). Additionally, with the complemented strains (*cPMI3191* and *cwaaE*), no invasion was observed at either timepoint.

**Figure 3:**
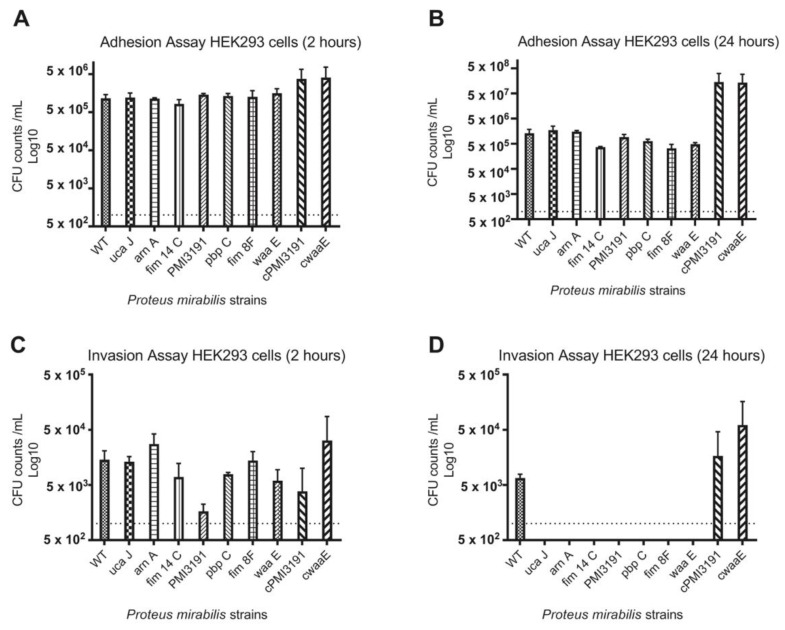
Adhesion and invasion of various *Proteus mirabilis* strains to and of, respectively, HEK293 kidney epithelial cells. (**A**) Adhesion after 2 h. (**B**) Adhesion after 24 h. (**C**) Invasion after 2 h. (**D**) Invasion after 24 h. The data are represented as the mean ± SD of triplicate samples of three independent experiments.

Next, we tested adhesion and invasion in HEK293 kidney epithelial cells: PM WT at 2 h p.i. was quantified at 1.2 × 10^6^ CFU/mL, while the number of adhered mutant strains ranged from 8.2 × 10^5^ CFU/mL to 1.6 × 10^6^ CFU/mL. There was no statistically significant difference between WT and the mutant strains with regard to their adhesion capabilities (Figure 3A). At 24 h p.i., adhesion tended to be lower for the mutants *fim14C*, *PMI3191*, *pbpC*, *fim8F*, and *waaE*, although again, no statistically significant difference in adhesion patterns was found (Figure 3B). The complemented strains (*cPMI3191* and *cwaaE*) showed more adherence to HEK293 cells at both 2 h and 24 h.

At 2 h, WT invaded the HEK293 cells at a concentration of 1.4 × 10^4^ CFU/mL, and although not statistically significant, PMI3191 and waaE displayed very low levels of invasion at 1.7 × 10^3^ CFU/mL and 6.0 × 10^3^ CFU/mL, respectively (Figure 3C). At 24 h p.i., invasion was only seen for WT (6.7 × 10^3^ CFU/mL), and none of the mutants invaded the cells (Figure 3D). However, after complementation of the knocked-out gene, invasion was apparent at 2 and 24 h for *cPMI3191* and *cwaaE*, with high levels of invasion after 24 h of infection.

**Figure 4:**
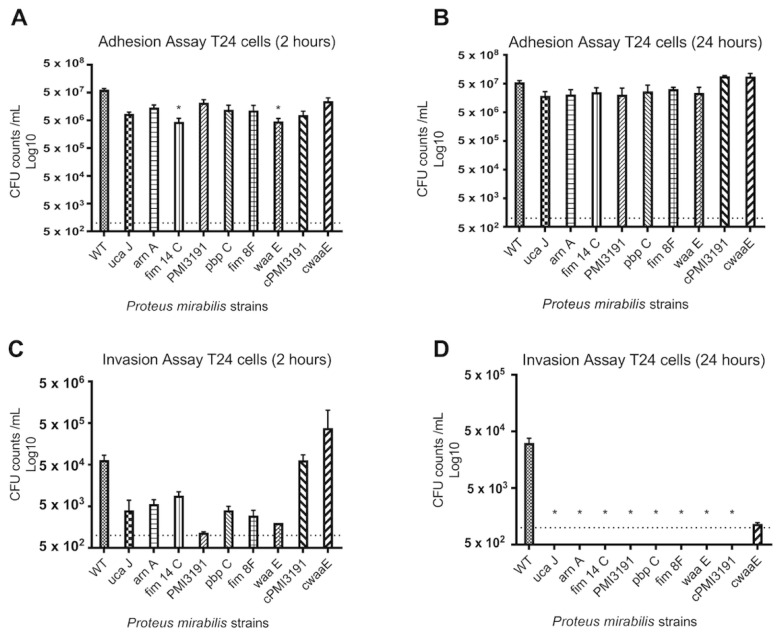
Adhesion and invasion of various *Proteus mirabilis* strains to and of, respectively, T24 bladder epithelial cells. (**A**) Adhesion after 2 h. (**B**) Adhesion after 24 h. (**C**) Invasion after 2 h. (**D**) Invasion after 24 h. The data are represented as the mean ± SD of triplicate samples of three independent experiments. Statistically significant differences are indicated by an asterisk (* *p* < 0.05).

We also tested invasion and adhesion in T24 bladder epithelial cells: WT showed significantly more adhesion at 2 h p.i. when compared to *fim14C* and *waaE* (Figure 4A). However, at 24 h p.i. levels of adhesion for MSs tended to be lower, but not statistically significant. Adhesion of the complemented strains (*cPMI3191* and *cwaaE*) to T24 cells exceeded adhesion levels of WT at 24 h (Figure 4B). At 2 h p.i. we could demonstrate invasion into T24 cells for all *PM* strains; however, MSs invaded at significantly lower levels when compared to WT. Only the complemented strains reached comparable levels of invasion after 2 h compared to WT (Figure 4C). Further, at 24 h p.i., no mutant strain of *PM* was capable of invading the bladder cells. However, after complementation, the invasion could be demonstrated for *cwaaE*, while no invasion was seen for *cPMI3191* when only the wildtype was able to invade T24 cells at 24 h (Figure 4D).

### 3.3. In Vivo Results

To further investigate the in vitro findings we tested *PM* WT’s and MS’s ability to adhere to and invade mouse tissue using an established CAUTI mouse model. Mice underwent inhalational anesthesia, and a 4 mm piece of a modified 24G PU catheter was implanted into the mouse bladder under ultrasound guidance on day 0. The next day (day 1), a bacterial solution containing 5 × 10^5^ CFU/mL in 50 μL PBS of one of the strains to be tested was percutaneously injected into the mouse bladder lumen using a 30G needle. On day 4 (48 h post infection), animals were anesthetized urine aseptically collected using a 30G needle under ultrasound guidance for subsequent quantification of planktonic bacteria via CFU counts as described above. Subsequently, all mice were euthanized.

**Figure 5:**
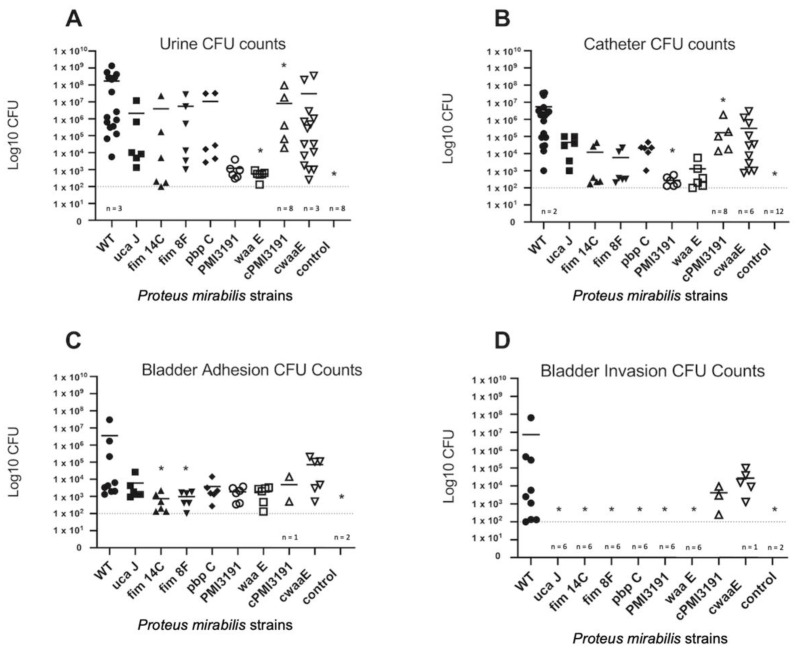
CFU counts of in vivo testing of the various PM strains. (**A**) Urine CFU counts (**B**) CFU counts of bacteria adhered to the bladder catheter. (**C**) CFU counts of bacteria that adhered to bladder cells. (**D**) CFU counts of bacteria that invaded into bladder cells. The solid bar represents the mean number of bacteria, and data are shown on a log10 scale. Each symbol represents the total CFU recovered from a single animal. We used the Kruskal–Wallis test (nonparametric one-way ANOVA). Statistically significant differences are indicated by an asterisk (* *p* < 0.05).

On day 4 of the experiment (48 h p.i.), the highest number of bacteria in urine were found for WT, while those for the MSs were significantly lower (Figure 5A). The lowest CFU counts were observed for *PMI3139* and *waaE* mutants, with the complementation of these genes resulting in adhesion levels returning to levels similar to WT. All control mice, injected with sterile PBS, yielded 0 CFU counts.

In terms of bacterial adhesion to catheters (Figure 5B), WT *PM* was found to have the greatest adhesion compared to the mutant strains. Both *PMI3139* and *waaE* mutants adhered at the lowest levels to catheters. The complementation of these genes resulted in adhesion levels similar to that of the WT strain. Catheters from the control did not have any adherent bacteria.

In terms of interaction with the urothelium (Figure 5C), the WT strain resulted in the greatest number of adherent bacteria compared to the mutant strains, which were all significantly reduced in their adhesion ability. The complementation of *PMI3139* and *waaE* resulted in a trend towards increased adhesion, which did not reach WT levels.

In terms of the number of bacteria that invaded the urothelium (Figure 5D), internalization was detected only for the WT strain, while none of the mutant strains were recovered from the bladder tissue. The complementation of *PMI3139* and *waaE* did rescue the loss of invasiveness, albeit at levels lower than the WT strain.

## 4. Discussion

*Proteus mirabilis* is a notoriously strong biofilm former and is therefore a frequent cause of urinary tract infections in individuals with indwelling foreign bodies within the urinary tract, such as bladder catheters, ureteral stents or nephrostomy tubes, and often leads to the encrustation of devices and/or infection stones [28]. To date, not much is known regarding the role of specific bacterial surface components in *PM* pathogenicity, especially the adherence to indwelling devices leading to biofilm formation and subsequent adhesion and invasion of uroepithelial cells. To address this gap in knowledge, the present work investigates the role of different bacterial components in mediating these key processes involved in *PM* pathogenicity using both in vitro and in vivo settings.

All mutants were found to have decreased adhesion to PU catheters. Considering that these experiments were performed in artificial urine, which has limited proteins, adhesion in this setting is mainly driven by charge and hydrophobicity characteristics rather than the direct interaction of adhesins with proteins deposited on the material surface [29]. Interestingly, the level of adhesion to the catheter surface was similar for WT and mutant strains at both the 4 h and 24 h timepoints. While one would expect greater adhesion levels of a given strain at a later timepoint, this may not be the case in the present study due to the charge-driven adhesion taking place in this environment, plateauing within the first few hours and not changing significantly as the bacteria transitioned from initial adhesion to colonization in the first 24 h. It is unlikely that the observed pattern is due to the loss of viability in planktonic bacteria at the later timepoint due to a higher pH, as *P. mirabilis* has been shown to prefer a higher pH to undergo adhesion processes. Furthermore, bacteria within a biofilm remain alive for at least several days in a higher-pH environment [30]. A similar pattern of adhesion to the same type of catheter was also observed in vivo, with the exception of the *PMI3191* and *waaE* mutants, which adhered to a lesser extent than the other mutants. Interestingly, lower numbers were also recovered from the urines of animals infected with these two mutants despite similar inoculation numbers, suggesting that lower adhesion may be the result of fewer bacteria being retained in the urinary tracts of these animals. That being said, reduced adhesiveness and bacterial numbers of these mutants in urine did not translate to the bladder tissue, as both adhesion and invasion levels were similar to the remaining mutants. This may suggest that despite lower numbers being retained in the urine, these mutants were more efficient at adhering to the bladder tissue. The fact that complementation of *PMI3191* and *waaE* resulted in adhesion and invasion levels similar to the WT strain suggests that changes in these key pathogenic steps were driven by changes in LPS structure, as these genes express LPS biosynthesis genes, and at least knocking out *waaE* resulted in significant changes in LPS banding patterns that were resolved by complementation.

None of the mutants were found invading the bladder tissue when the animals were sacrificed 48 h post-infection. To better understand the adhesion and invasion patterns of WT and mutant strains, these key processes were assessed in three different cell lines representing the bladder and kidney. Overall, we found adhesion and invasion patterns to differ between the different cell types. While adhesion was somewhat affected for some mutants at the 2 h timepoint for all cell types, no differences were observed at 24 h post-infection. In contrast, none of the mutants were found to invade A498 cells at either timepoint, while some initial invasion was evident for HEK293 cells that were lost by 24 h. A similar pattern was also observed for T24 bladder cells, which suggests that a lack of invasion in the mice may be due to an inability of the mutants to invade bladder cells. In terms of the role for LPS structure in these processes, complementation of *PMI3191* and *waaE* resulted in adhesion levels that were greater than the corresponding mutant and, in some cases, similar to WT. The fact that complementation of *PMI3191* and *waaE* resulted in the rescue of the invasion phenotype for both HEK293 and T24 cells suggests that the LPS structure plays a role in mediating the invasion of uroepithelial cells by *PM*. Changes in LPS glycosylation such as those expected from the loss of a glycosyltransferase (i.e., PMI3191) have previously been shown to affect the invasiveness of *Shigella flexneri*, with strains with increased glycosylation showing enhanced invasion [31]. Similarly, alterations of the LPS inner core were shown to significantly affect the invasiveness of uropathogenic *E. coli*, which may explain the loss of invasion of the *waaE* mutant [32]. That said, this same role was not observed in A498 cells, as complementation of *PM319*1 and *waaE* did not rescue the invasiveness in this cell type, suggesting that a role for *PM* LPS in invasion may be cell-dependent. This certainly requires further investigation.

Fimbriae have been the subject of continued investigation in *PM* virulence, and their crucial role in infection pathogenicity by aiding adhesion and aggregation in vivo has been demonstrated [9]. Fimbriae-deficient strains have shown significantly decreased infectivity when compared to the wildtype, with decreased kidney and bladder colonization and adherence [33]. These findings are in accordance with our results, as *ucaJ, fim8F,* and *fim14c* mutants showed substantially lower CFU counts in the adhesion assays at both timepoints in the majority of experiments. *UcaJ* is a uroepithelial cell adhesin (UCA) mutant, and UCA fimbriae have been identified to play a critical role in the attachment of *PM* to epithelial cells of the urinary tract [12]. Pellegrino et al. observed that an *ucaA* mutant demonstrated diminished adhesion to the urothelium, compared to wildtype [34]. Their results suggested that the urothelial cell adhesin of *PM* may be a crucial factor in kidney colonization, as well as the onset and propagation of CAUTI.

ArnA is a bifunctional polymyxin resistance protein that is required for the modification of lipid A in LPS, and this modification leads to bacterial resistance to cationic antimicrobial peptides of the host immune system, as well as resistance to certain antibiotics, such as polymyxin [17]. These proteins have seen to be conserved across many different Gram-negative bacteria, including *E. coli* and *Pseudomonas aeruginosa* [17]. Another study looking at the disruption of the *arnA* gene in a *PM* CAUTI mouse model specifically indicated that it leads to a decrease in bacterial fitness in the urinary tract, as well as the spleen, which may be indicative of bacteremia [11]. CFU counts of *arnA* mutant bacteria that adhered to kidney epithelial cells were comparable to those of WT, but there were substantially lower CFU counts in the bladder cells when compared to WT, indicating that *arnA* plays a role in the interaction of *PM* with the uroepithelium.

Penicillin-binding proteins (PBPs) are high-molecular-weight proteins that are attributed to crucial steps in peptidoglycan synthesis of Gram-negative bacteria [18]. *PM* has been identified to possess PBPs, and the *pbpC* mutant strain used in this study lacks enzymatic abilities associated with peptidoglycan synthesis [19]. Interestingly the *pbpC* mutant showed the same patterns in the adhesion and invasion assays as mutants in fimbriae, suggesting that changes in peptidoglycan structure play a role in these important processes.

Collectively, the present work provides a broad overview of bacterial surface structures critical for different critical steps in the pathogenicity and the overall infectious process of *PM*, including the adhesion to indwelling medical devices, as well as the adhesion and invasion of urinary tissue cells and the tissue itself. These data suggest a role for all surface components studied in these key processes. Considering that no mutant resulted in the complete loss of tissue adhesion and invasion indicates that these processes likely involve the dynamic interplay between multiple components. Future experiments should focus on utilizing *PM* strains with mutations on multiple key components to determine whether targeting more than one results in the loss of infection. To that end, the data presented herein represent a critical first step towards the development of targeted therapies to block key steps in the pathogenicity of PM and prevent associated morbidity and mortality.

## Figures and Tables

**Table 1:** *Proteus mirabilis* mutants.

PMID	Gene Name	Gene/Protein Function
PMI0532	*ucaJ*	Fimbria
PMI1045	*arnA*	LPS formation
PMI2998	*fim 14C*	Fimbria
PMI3191	*PMI3191*	Glycosyl transferase
PMI1850	*pbpC*	Peptidoglycan synthesis
PMI1464	*fim 8F*	Fimbria
PMI3166	*waaE*	LPS formation

## Data Availability

The data presented in this study are available upon reasonable request from the corresponding author via dirk.lange@ubc.ca.

## Supplementary Materials

The following supporting information can be downloaded at: https://www.mdpi.com/article/10.3390/pathogens12040509/s1, Figure S1: LPS of the 10 different PM strains.
