# Supplementary material for: Role of Bacterial Surface Components in the Pathogenicity of Proteus mirabilis in a Murine Model of Catheter-Associated Urinary Tract Infection
Source: Pathogens. 2023 Mar 24;12(4):509. doi: 10.3390/pathogens12040509 (PMC10143806; doi:10.3390/pathogens12040509)
Supplement: Supplementary file 1 [file pathogens-12-00509-s001.zip › pathogens-2212176-supplementary.pdf]

Supplementary Materials:

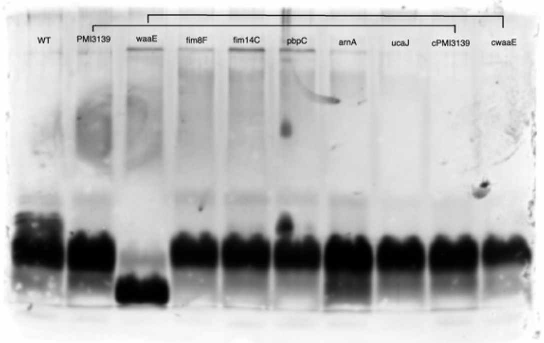

Figure S1. LPS of the 10 different *PM* strains separated on a 15% SDS-polyacrylamide gel.
